# Accounting for retest effects in cognitive testing with the Bayesian double exponential model via intensive measurement burst designs

**DOI:** 10.3389/fnagi.2022.897343

**Published:** 2022-09-26

**Authors:** Zita Oravecz, Karra D. Harrington, Jonathan G. Hakun, Mindy J. Katz, Cuiling Wang, Ruixue Zhaoyang, Martin J. Sliwinski

**Affiliations:** ^1^Department of Human Development and Family Studies, Pennsylvania State University, University Park, PA, United States; ^2^Institute for Computational and Data Sciences, Pennsylvania State University, University Park, PA, United States; ^3^Center for Healthy Aging, Pennsylvania State University, University Park, PA, United States; ^4^Department of Neurology, Pennsylvania State University, Hershey, PA, United States; ^5^Department of Psychology, Pennsylvania State University, University Park, PA, United States; ^6^Department of Neurology, Albert Einstein College of Medicine, Bronx, NY, United States; ^7^Department of Epidemiology and Population Health, Albert Einstein College of Medicine, Bronx, NY, United States

**Keywords:** retest learning, measurement burst design, double negative exponential model, subtle cognitive decline, Bayesian multilevel modeling

## Abstract

Monitoring early changes in cognitive performance is useful for studying cognitive aging as well as for detecting early markers of neurodegenerative diseases. Repeated evaluation of cognition via a measurement burst design can accomplish this goal. In such design participants complete brief evaluations of cognition, multiple times per day for several days, and ideally, repeat the process once or twice a year. However, long-term cognitive change in such repeated assessments can be masked by short-term within-person variability and retest learning (practice) effects. In this paper, we show how a Bayesian double exponential model can account for retest gains across measurement bursts, as well as warm-up effects within a burst, while quantifying change across bursts in peak performance. We also highlight how this approach allows for the inclusion of person-level predictors and draw intuitive inferences on cognitive change with Bayesian posterior probabilities. We use older adults’ performance on cognitive tasks of processing speed and spatial working memory to demonstrate how individual differences in peak performance and change can be related to predictors of aging such as biological age and mild cognitive impairment status.

## Introduction

Accurate and sensitive measurement of cognitive change is required to advance the understanding of normative cognitive aging and improve the detection of the subtle cognitive changes that are associated with the preclinical stages of neurodegenerative diseases, such as Alzheimer’s disease. Although cumulative cognitive change over the course of decades is quite robust, the amount of change expected over a year or two is quite subtle, even in the case of prodromal disease (Baker et al., 2016). Traditional methods relying on infrequent lab-based assessment of cognitive performance make it difficult to differentiate changes due to cognitive aging, progression of neurodegenerative disease, and the possible effects of interventions designed to improve or slow decline in cognitive function. A major challenge to detecting subtle cognitive change is the presence of retest, or practice effects, which refer to the ubiquitous finding that performance on cognitive tests improves with repeated testing.

**FIGURE 1:**
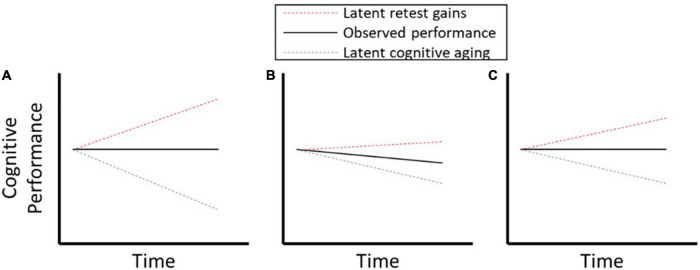
**(A–C)** Illustration of retest effects confounding measurement of cognitive decline.

Although widely recognized as biasing longitudinal estimates and intervention effects, there is no consensus on best methods to address retest effects (Jones, 2015). Indeed, it is extremely difficult to disentangle retest related effects from other sources of change (e.g., aging, disease progression, and interventions) using data from conventional longitudinal designs that consist of repeated single-shot assessments, usually spaced over long time intervals. Figure 1 illustrates this point by depicting hypothetical longitudinal data in which observed performance (black line) reflects a mixture of two latent processes, retest related gains (red line) and aging-related declines (blue line). Panel A shows a case in which change in performance is flat, where the stability is a product of retest related gains offsetting aging-related decline. Comparing manifest performance (black lines) in Figure 1, panels B and C suggests that the former exhibited more cognitive decline and that neither exhibited evidence of improvement in cognitive performance that could be due to retest effects. However, the underlying latent aging effects show equivalent longitudinal decrements in Panels B and C, but differential latent retest effects.

This example illustrates two important points. First, processes that drive retest effects may be operating even if manifest performance shows no improvement or even a decline. That is, one cannot take the absence of overt performance gains as evidence that retest effects are absent. Moreover, even in the presence of manifest decline in cognitive performance, retest effects may be a significant confound that obscures important individual differences. Second, failure to accurately characterize and account for retest gains could add considerable noise and bias when testing for the effects of interventions, biological markers of aging or disease progression, or other exposures (e.g., stress, environmental toxicants) on cognitive trajectories.

Conventional longitudinal designs place significant constraints on approaches for disentangling retest effects from other types of change. The use of a control group which receives their first exposure to a cognitive test at follow-up may be useful for estimating bias in the group averages but cannot assist in correcting for retest effects at the individual level. Statistical control procedures that involve covarying for the number of retest assessments are susceptible to bias and are especially sensitive to assumptions regarding the presence of age-cohort effects (Hoffman et al., 2011). To overcome these limitations, our approach utilizes a measurement burst design (Sliwinski, 2008) which consists of closely spaced “bursts” of repeated measurements which are repeated over longer intervals. This type of intensive longitudinal design (ILD) permits modeling of retest effects using repeated administrations over a short interval within bursts (e.g., daily) to render long-term retest effects negligible and to model long-term trends using measurements bursts repeated over longer intervals (e.g., annually) across bursts (Sliwinski et al., 2010; Rast et al., 2012). In a proof of concept, Munoz et al. (2015) fit a non-linear multilevel model to measurement burst data to disentangle short-term retest effects from long-term declines in asymptotic performance.

We propose a psychometric cognitive process model, the Bayesian double exponential model (BDEM) to disentangle retest learning effects from longitudinal changes in asymptotic performance. The BDEM allows parameterizing performance in terms of distinct retest features including learning rate (how quickly someone reaches peak performance), retest gains (how much overall improvement is observed), peak (asymptotic) performance, and warm-up effects that occur at the beginning of follow-up bursts. Once practice effects are accounted for, we can link individual differences in peak performance and changes in peak performance to person-level indicators such as age and mild cognitive impairment (MCI) status.

While the primary aim of BDEM is to disentangle learning features from peak performance (with the goal of modeling asymptotic change over time), each model parameter may also be of interest for understanding the dynamics of cognitive change. For this reason, we also quantify individual differences, in a multilevel framework, not only in terms of peak performance and changes therein, but also for example in terms of learning rate, and intra-individual variability in performance, and test whether these are linked to cognitive aging (Lövdén et al., 2007) or MCI status (Cerino et al., 2021).

Compared to earlier work with the double negative exponential model, such as in Sliwinski et al. (2010), Broitman et al. (2020), our approach casts the double negative exponential model in a multilevel Bayesian statistical framework, which has two main advantages. First, it allows for all double exponential parameters to be person-specific and be regressed on person-level predictors in a single step analysis, this way improving estimation accuracy. Second, it allows for a more nuanced inference in terms of person-specific characteristics, for example the risk of cognitive decline can be articulated in terms of individual specific probabilities, as illustrated later in the paper.

In the current study we analyzed data from the Einstein Aging Study (EAS; Zhaoyang et al., 2021; Katz et al., 2021), a longitudinal study that included annual conventional assessments and ambulatory assessment bursts in a racially diverse, systematically recruited community dwelling cohort of older adults (age 70+). We evaluated the descriptive adequacy of the BDEM to EAS data obtained from high frequency cognitive assessments completed by participants using mobile devices in naturalistic settings. We also examined whether BDEM parameters, such as asymptotic performance, change in asymptotic performance, learning rate, and intra-individual variability, differentiated among individuals across different ages and MCI status.

## Materials and methods

### Study design and procedure

Data were drawn from the ongoing EAS, a prospective, longitudinal study of risk factors for MCI and dementia. Systematic random sampling from New York City Registered Voter Lists for Bronx County was used to recruit participants. Further screening of participants was conducted via telephone to ensure that participants met the study inclusion criteria: English-speaking, community-residing, ambulatory, and aged over 70 years. Exclusion criteria were: significant hearing or vision loss, current substance abuse, severe psychiatric symptoms, chronic medicinal use of opioids or glucocorticoids, treatment for cancer within the past 12 months, and diagnosis of dementia. All participants provided written informed consent and the study was approved by the Albert Einstein College of Medicine Institutional Review Board.

**FIGURE 2:**
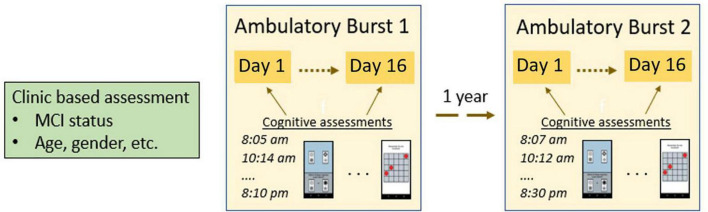
Illustration of a measurement burst design with two bursts.

Figure 2 shows an illustration of the overall measurement burst design deployed in the EAS project. Each year participants completed a combination of clinic-based assessments and ambulatory ecological momentary assessments (EMA). After telephone screening, eligible participants were invited to attend two in-person clinic-based assessments. The first assessment day included completing self-report questionnaires and neuropsychological assessment. The second assessment day included a 1.5-h training session on how to use the study-provided smartphone and complete the EMA portion of the study. Participants who were assessed between March and June 2020 completed modified versions of these assessments and training remotely via telephone and received the study smartphone via a package delivery service.

The ambulatory burst component of the study took place in participants’ natural environments. While participants went about their daily activities, they completed six brief assessments (up to 5 mins each) during their typical waking hours, over a period of 16th days−these assessments together formed a “burst.” The assessments included brief self-report questions as well as the cognitive assessments. The protocol included a self-initiated wake-up assessment, a self-initiated end-of-day assessment, and four quasi-random “beeped” assessments that participants received a notification from the study phone to complete. The beeped assessments were schedule approximately 3.5 h apart, and times varied across the days of the week. After the ambulatory burst period, participants returned the study smartphone at a third clinic visit and the data were downloaded from the phone.

In the present study we analyzed baseline demographic and MCI status data, as well as cognitive performance data from Burst 1 and Burst 2, that were collected between May 2017 and June 2020. The sample consisted of 318 adults, of which 53.8% (*n* = 171) completed both bursts, while the remaining participants had only baseline (Burst 1) data. Of the 147 participants who did not have follow up (Burst 2) data, 31.3% (*n* = 46) had not yet been contacted for follow up, 24.0% (*n* = 35) had chosen to not complete the EMA component of the study, 4.0% (*n* = 6) had missing or unusable data on the smartphone, 8.2% (*n* = 12) were unable to participate due to illness or death, and 36.7% (*n* = 54) were withdrawn. Characteristics of the sample are provided in the “Results” section.

### Measures

#### Demographics

Participants self-reported demographic details via questionnaire, including age in years, sex (male/female), race and ethnicity (White/Black/Hispanic White/Hispanic Black/Asian/more than one race), and education (years in school).

#### Mild cognitive impairment status

As part of their participation all participants underwent neuropsychological assessment to determine their cognitive status. The neuropsychological assessment included measures of memory, executive function, attention, language, and visuospatial ability. Free recall from the Free and Cued Selective Reminding Test (Buschke, 1984) and delayed recall of the Benson Complex Figure (Possin et al., 2011) assessed memory function; Trail Making Test – Part B (Reitan, 1958) and Phonemic Verbal Fluency (Tombaugh et al., 1999) assessed executive function; Trail Making Test – Part A (Reitan, 1958) and WAIS-III Digit Span (Wechsler, 1987) assessed attention; Multilingual Naming Test (Ivanova et al., 2013) and Category Fluency (Monsch et al., 1992) assessed language; and immediate recall of Benson Complex Figure (Possin et al., 2011) and WAIT-III Block Design (Wechsler, 1987) assessed visuospatial function. MCI status was classified algorithmically using criteria from described in Jak et al. (2009) and described in detail in Katz et al. (2021). Briefly, criteria included: (a) impaired scores on two measures of the same cognitive domain; or (b) one impaired score in three out of five cognitive domains; or (c) having functional decline assessed by the Instrumental Activities of Daily Living Scale (Lawton and Brody, 1969). Impairment was defined as scores >1 SD below age-, sex-, and education- adjusted normative mean.

#### Symbol search task

**FIGURE 3:**
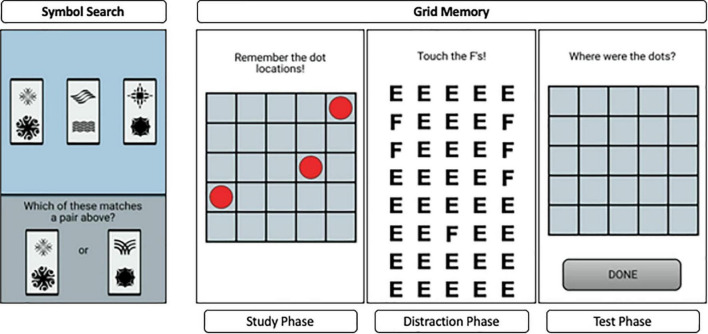
An example trial from the symbol search task **(top)** and the grid memory task **(bottom)**.

The symbol search task, shown on the left side of Figure 3, measures processing speed. In the current study, on each trial of the task, participants saw three symbol pairs at the top of the screen and two symbol pairs at the bottom of the screen. They were instructed to match as quickly and accurately as they could one of the two pairs presented at the bottom to one of the three pairs at the top. Participants completed 11 trials per session. We analyzed daily aggregates of response times on correct trials with the BDEM.

#### Grid memory task

Grid Memory is a free recall paradigm that assesses spatial working memory, shown on the right side of Figure 3. This task in the current study involved a brief study phase, during which 3 dots are presented at random locations on a 5 × 5 grid for 3 s, an 8-s letter-cancelation distractor phase, followed by free recall of locations occupied by dots during the study phase. The free recall phase required participants to touch the locations in an empty 5 × 5 grid where the 3 dots were presented initially. Participants completed two trials that incorporated all three phases per session. The outcome of interest for this task was the Euclidean distance between the location of the incorrect dot to the correct grid (0 if correct). This gave partial credit based on the deviation of the recalled compared to the correct dot locations. We refer to this error distance as “units of error” from here on.

### Data analysis with the double exponential model

First, we start by specifying a negative exponential model for repeatedly administered cognitive performance data close in time. This model can characterize change in performance in terms of four parameters: learning rate, retest gain, peak performance and intra-individual variability. By disentangling the latent processes in observed performance, the negative exponential model separates *how much* learning occurs (retest gain) from *how fast* the learning occurs (learning rate). The learning curve is characterized by an exponential shape, which is supported by studies on learning (see, e.g., Heathcote et al., 2000), as well as studies on aging (see, e.g., Sliwinski et al., 2010). These curves will be derived for every person to accurately dissociate learning from the person-level overall peak performance.

**FIGURE 4:**
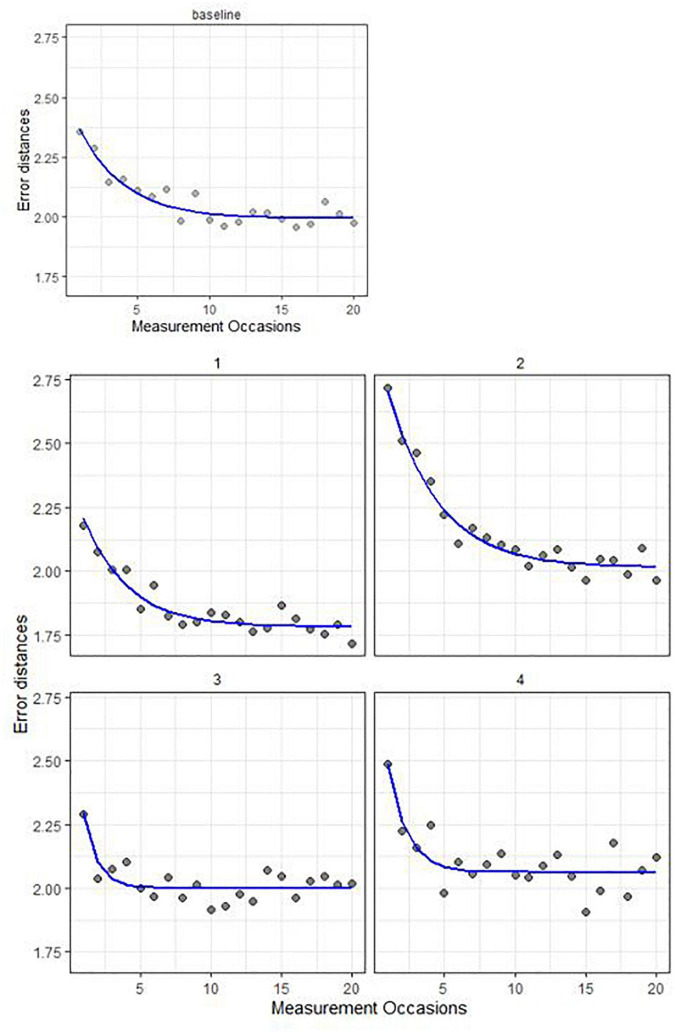
Five synthetic participant’s data (gray dots) and model fit (solid line).


Consider a study that only has one burst of measurements. The top row of Figure 4 shows a graphical representation of the negative exponential model (solid line) fit to a sequence of a simulated cognitive performance measure (indicated by dots), which in our case was either error distance or reaction time. We will refer to this participant as the “baseline” for later comparisons. At the start of the burst, their errors are distinctly higher than near the end; that is, the participant shows evidence of learning across sessions in a measurement burst. This improvement is modeled through a negative exponential function, which is parametrized as follows:

(1) $$Y_{t\,i}=a_{i}+g_{i}\times \exp\left[-r_{i}\times M_{t\,i}\right]+e_{t\,i}$$

The cognitive performance data over sessions *t* from an individual *i* is denoted as *Y*_*ti*_. Parameter *a_i_* stands for the person’s asymptotic or peak performance (best performance given unlimited practice), which was set to 2.00 in the current example shown in the top row of Figure 4. The gain in performance across measurements is quantified by *g_i_*, which roughly corresponds to the height of the exponential (set to 0.50 in this example). The learning rate is captured by *r_i_* (set to 0.30), the steepness of the exponential curve across measurements (with measurement occasions denoted as *M*_*ti*_) in the study. Finally, *e*_*ti*_ is a time-and-person-specific error term, with mean zero and standard deviation σ_*e*,*i*_ (set to 0.05), where σ_*e*,*i*_ captures the within-person variations (i.e., intraindividual variability) across trials.

The bottom two rows of Figure 4 shows four additional synthetic persons’ data and model fit, each with one parameter different from the “baseline” in the top row. The participant in the left panel of the second row has better peak performance (less error): their asymptote settles at 1.75 instead of 2.00. The right panel of the second row shows a participant with a higher gain parameter across trials compared to the baseline person in the top row (*g_i_* is 1.00 instead of 0.50); the exponential starts out higher compared to baseline. The bottom left panel depicts a faster learning rate (steeper exponential slope; *r*_*i*_ is set to 0.60 instead of 0.30) than the baseline; and it reaches peak performance faster (given the same amount of gain). Finally, the bottom right panel shows a participant with greater intra-individual variation (σ_*e*,*i*_), as indicated by the more scattered dots around the fitted model (set to 0.10 instead of 0.05). Simulation analyses showed good recovery of these parameters with 10 data points per person, in terms of at least 95% of the simulated values falling in the estimated 95% credible interval of their corresponding parameter estimates.

The double negative exponential model extends the previously introduced negative exponential model by considering retest gains across bursts as well. It is specified as:


(2) $$Y_{t\,i}=g_{i}\times \text{exp}\,\left[-r_{i}\times M_{t\,i}\right]+I\left(B_{t\,i}>1\right)\times g_{i}^{*}\times \exp\left[-r_{i}\times T_{t\,i}\right]+e_{t\,i}+a_{i}+{△}_{i}\times \left(B_{t\,i}-1\right)$$

Parameters *g_i_* and *r*_*i*_ again represent gain and learning rate, as in Eq. (1), but now we have two sets of them: one (*g_i_*, *r_i_*) set to capture *continuous learning throughout the study* [much like in Eq. (1) when we only had one burst], shown in the first line of the Eq. (2), and a second ($g_{i}^{*}$, $r_{i}^{*}$) set that represents *warm-up processes after the first burst* [i.e., *I*(*B*_*ti*_ > 1)] – with gain and learning rate parameters denoted by an asterisk (*), as shown in the second line of Eq. (2). This warm-up effect also has an exponential functional form, and it operates on the measurements nested within a burst (denoted with *T*_*ti*_). Similarly to Eq. (1), *e*_*ti*_ again represents a time and person-specific error term, with its standard deviation σ_*e*,*i*_ quantifying the intraindividual variability in performance across all trials.

Most importantly, as shown in △_*i*_ × (*B*_*ti*_−1) of Eq. (2), we are now modeling the change in asymptotic (peak) performance between bursts. This is accomplished by parameter △_*i*_, which adjusts the asymptotic performance (*a*_*i*_), by some magnitude of change in every burst following the first one. We then investigate individual differences in these key parameters by adding covariates such as age, sex, and MCI status to the model.

**FIGURE 5:**
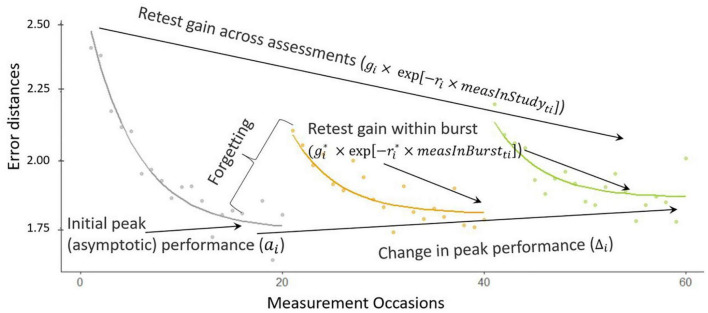
Illustration of the double negative exponential model.

Figure 5 shows a graphical representation of the double negative exponential model fit to cognitive performance measures (error distances in this example, displayed as dots) over trials *t* from a synthetic individual *i*, over three bursts (note that in the dataset analyzed later there are only two bursts, but we display three here for illustration purposes of the general approach). We can see that in the beginning of the study, there are more errors than at the end of the study, while also within each burst the first error rates are higher than the rest. We also observe retest gain across assessments: a learning process across the whole study period (here parameter *g_i_* quantifies person *i*’s gain across all measurements in the study, and *r_i_* represents their corresponding learning rate). Also, in each burst after the first, there is a warm-up gain, a within-burst learning process with gain $g_{i}^{*}$ and learning rate $r_{i}^{*}$ parameters. In the first burst of data (over measurement occasions 1−20), the asymptote represents the person-specific initial peak performance, which becomes worse with every burst in this example (higher asymptotes correspond to more error). The amount of change in peak performance is quantified by △_*i*_. As can also be seen in Figure 5, due to the retest gain across assessments and within burst, there seems to be an improvement in performance (decrease in error distances overall across the study). However, if we look at the long-term change in terms of the peak performance parameter of the proposed model (i.e., change in asymptote), there is in fact an incremental decline in performance, manifested through an increase in errors (i.e., worsening peak performance across bursts).

The warm-up effect represents an expected decrease in performance from the peak performance of a prior burst to the initial performance at its follow-up burst. It is an important process to model as this decrease may not reflect true “cognitive decline” and could instead represent some “forgetting” of testing procedures (see also in e.g., Dutilh et al., 2009). Our proposed modeling approach captures participants recovering their previous gains overlaid on their continuous improvement.

Modeling cognitive performance in terms of the person-specific double negative exponential parameters can help us capture retest effects and isolate them from other cognitive performance indicators. Investigating possible sources of individual differences (e.g., age, MCI status) in these cognitive parameters (i.e., learning rate, change in peak performance, etc.) can help us learn about processes related to retest effects and cognitive decline. In summation, our proposed model represents a cognitive psychometric approach to interpreting cognitive performance data. This model will require a nuanced and flexible statistical framework for inference. We chose a multilevel Bayesian framework (Gelman and Hill, 2007) for implementing the double negative exponential model, discussed next.

#### The multilevel specifications of the double exponential model

In our proposed modeling approach, all double negative exponential parameters, *a_i_*, △_*i*_, *g*_*i*_, *r_i_*, $g_{i}^{*}$, $r_{i}^{*}$, and σ_*e*,*i*_ were allowed to be person-specific and are pooled together via group-level (population) distribution. This represents a standard multilevel modeling approach (Raudenbush and Bryk, 2002) that has been proven useful for improving estimation accuracy, as it allows for the person- and group-level trends to simultaneously inform each other. Moreover, we also aim at identifying possible sources of individual differences in these parameters. Therefore, we regress person-specific cognitive performance characteristics (e.g., peak performance, change in peak performance, etc.) on a set of predictors, such as age and MCI status. Note that these cognitive performance characteristics are themselves model parameters, therefore they are estimated with error. We use a one-step approach to regress them on predictors to avoid generated regressor bias (Pagan, 1984).

More specifically, in our multilevel specification of the DNE, the peak performance, *a_i_*, changes in peak performance, △_*i*_, and intra-individual variation in performance, σ_*e*,*i*_, and learning rates (*r_i_*, $r_{i}^{*}$) have group-level (population) distributions, the means of which are decomposed into products of predictors and regression coefficients. For example, the person-specific peak performance, *a_i_*, parameters are assumed to follow a normal distribution, parametrized as:


$$a_{i}∼N\,\left({\text{x}}_{i}\,\beta_{a},\sigma_{a}\right),$$

where ***x**_i_* is a vector with a set of person-specific predictors such as sex (i.e., male or female) and MCI status, and with 1 as its first element (for the intercept). Vector ***β**_a_* contains the corresponding regression coefficients. Specifically, the first coefficient of ***β**_a_*, that is β*_a, int_*, takes the role of an intercept and quantifies the group (population)-level peak performance, while the rest of the regression coefficients correspond to the effects of the predictors in **x**_*i*_. In the analyses below we used age at baseline, MCI status, sex, and years of education as predictors. For example, regression coefficient β*_a, age_* quantifies the association between peak (asymptotic) performance and age at baseline, regression coefficient β*_a, MCI_* quantifies the association between peak performance and MCI status, regression coefficient β*_△, MCI_* quantifies the association between change in peak performance and MCI status, and so on. Parameter σ*_a_* quantifies residual variation in standard deviation units– that is individual differences remaining after the predictors are accounted for.

In the analyses below, similar specifications were made for △_*i*_, *r_i_*, $r_{i}^{*}$, and σ*_e,i_*, as well. The gain parameters were also made person-specific and assigned group-level distributions: *g*_*i*_∼*N*(μ_*g*_,σ_*g*_), where μ_*g*_ is the group-level mean gain across bursts, representing the average rate of gain in the sample throughout the study. The warm-up gain parameter $g_{i}^{*}$ is assigned a group-level distribution that follows the same logic. However, these gain parameters were not regressed on person-level predictors the same way as the other parameters, as we did not expect them to be meaningfully related to our chosen set of predictors.

Finally, note that we are not specifying any particular correlation structure on the person-specific parameters (i.e., random effects); we are not constraining the correlation to be 0. All parameters, including regression coefficients, negative exponential model parameters and variances are estimated in a Bayesian framework, introduced next.

#### Casting the multilevel double exponential model in a Bayesian framework

In the Bayesian framework model parameters are thought of as random variables that have their own probability distributions. Bayesian modeling focuses on the estimation of posterior probability distributions (i.e., posteriors) based on available data (interpreted through a likelihood function) and prior probability distributions (i.e., priors) on the model parameters. Prior probability distributions are mathematical summaries of any already existing information on the model parameters. All inference is conditional on the priors, and they need to be specified before seeing the data to genuinely reflect the already existing information available on the parameters. The prior distributions for this study were chosen to be minimally informative, reflecting only the plausible, theoretical range of the parameters. This involved truncating distributions to match the parameter’s range. For example, given that reaction times cannot be negative, peak performance of RT also cannot be negative, therefore its prior was truncated at 0 so that it cannot take on negative values.

Population means were given a prior that was distributed normally with 0 mean and standard deviation 10, truncated at 0 for across study (i.e., across bursts) and within-burst gain parameters, such as:


$$\mu_{g}∼N\,\,\left(0,10\right)\,\text{and}\,\mu_{g*}∼N\,\left(0,10\right).$$

Regression coefficients (except for intra-individual standard deviation) were given the same priors, for example:


$$\beta_{a,a\,g\,e}∼N\,\left(0,10\right),$$

was specified for the coefficient linking asymptote (peak performance) and age. For the intra-individual standard deviation, we chose a somewhat tighter normal distribution with standard deviation equal to 1 to reflect that the likely range of this parameter was between 0 and 1, for example as:


$$\beta_{\sigma_{e},a\,g\,e}∼N\,\left(0,1\right),$$

for the association between intra-individual standard deviation and age.

The group-level standard deviation parameters that reflect the heterogeneity across individuals were truncated to be on the positive real line and were specified as:


$$\sigma_{r}∼N\,\left(0,10\right),$$

where σ_*r*_ could be substituted with σ_r*_, σ_*g*_, σ_g__*_, σ_*a*_, and σ_δ_. The standard deviation of the intra-individual standard deviation was specified as:


$$\sigma_{\sigma_{e}}∼N\,\left(0,1\right),$$

with range again truncated to the positive real line.

### Implementation of double exponential model

The Bayesian double negative exponential model was implemented in Stan (Stan Development Team, 2022) called from R via rstan (Stan Development Team, 2020) – the Rscript for the estimation is available via OSF^1^.

The results below were based on 60,000 samples from the posterior probability distributions of each model parameter. Specifically, we ran 6 parallel chains drawing 12,000 samples each, from which 2,000 per chain were discarded as warm-up iterations, resulting in 60,000 total iterations for each parameter. Sampling was performed via Markov chain Monte Carlo (MCMC) algorithms implemented in Stan. We checked MCMC sampling quality by calculating effective sample size (ESS) and $\hat{R}$ statistics. ESS quantifies the number of independent pieces of information in the posterior distribution and should be at least 100, but preferably around 1,000 to get reliable interval estimates. The $\hat{R}$ statistic is indicative of convergence of the sampled values, and values above 1.1 signal issues with convergence (Gelman et al., 2013). In our analyses, the ESSs for all parameters were above 100, and 98% of ESSs were also above 1,000 and all $\hat{R}$s were below 1.1.

#### Model fit

##### Symbol search task

**FIGURE 6:**
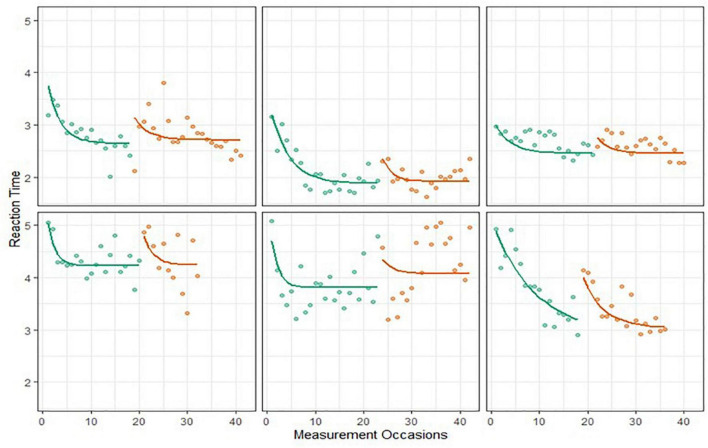
Six Einstein Aging Study (EAS) participants’ symbol search data and predicted BDEM trajectories.

We calculated the R^2^ statistic to quantify the proportion of the variance in reaction times explained by the BDEM. For the current dataset the R^2^ was 0.89, which supports a good fit of the model for the data. We also explored model fit visually by plotting model predicted trajectories over the data points, for every person, and concluded that the model followed the characteristics of the data satisfactorily. Specifically, we looked at whether the model predicted trajectory mimics the most important characteristics of the person-level data, which were whether (1) the height of the exponential curve overlaps with the first couple of observed data points, (2) the asymptote of the exponential curve overlaps with the best performances, and (3) the change in performance across the observations has an exponential shape. As an example, Figure 6 shows 6 persons’ raw data (dots) and model predicted trajectories, these were chosen to give representative illustration of the overall trends of the data.

##### Dot memory task

**FIGURE 7:**
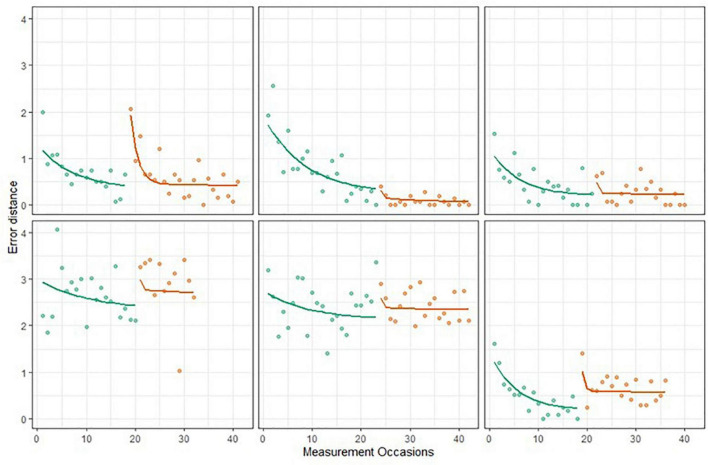
Six EAS participants’ grid memory data and predicted Bayesian double exponential model (BDEM) trajectories.

The BDEM showed sufficient fit to the grid memory data, with R^2^ = 0.76, and predicted trajectories showing acceptable patterns; see Figure 7 for examples. However, we note that the fit of the BDEM was not as ideal for this data as for the symbol search data^2^.

## Results

We analyzed data for 318 participants, from which 171 completed both bursts, while the remaining participants had only Burst 1 data. The mean age of the sample at baseline was 77.45 (4.83) years and 67% were female (*n* = 104 male, and *n* = 214 female). The sample was racially and ethnically diverse with 45.9% (*n* = 146) identified as non-Hispanic Whites, 39.9% (*n* = 127) as non-Hispanic Blacks, 9.7% (*n* = 31) as Hispanic Whites, 2.8% (*n* = 9) as Hispanic Blacks, 1.3% (*n* = 4) as Asian, and 0.3% (*n* = 1) as more than one race/ethnicity. The mean education of the sample was 14.98 (3.55) years. On that basis of the neuropsychological assessment and criteria described above, 31.8% (*n* = 101) participants were classified as having MCI at baseline. There was no significant difference between those who completed both bursts and those with only Burst 1 in terms of age, years of education, race/ethnicity, or sex. But the group with only Burst 1 data was significantly more likely to be classified as MCI at Burst 1 (40.41% v. 24.42%, *p* = 0.003; please see table with all comparisons on the paper’s OSF site). However, the BDEM mixed effects models we used can handle the missing data under the assumption that the data are missing at random (MAR). That is, the missing data process may depend on the predictors such as MCI status, covariates and the observed EMA cognitive outcomes at Burst 1. The only requirement for the missing data process is that conditional on MCI, covariates and Burst 1 EMA cognitive data, the missing data at Burst 2 must be independent of the unobserved Burst 2 cognitive performance.

### The symbol search task

We note that we decided to scale the response times in seconds to keep the above specified prior settings in the estimation algorithm the same for the symbol search and for the grid memory task data.

We did an initial data exploration by comparing the differences in Burst 1 and Burst 2 manifest performances (i.e., no BDEM). For every person, we calculated their average Burst 1 and Burst 2 reaction times, and created a difference score based on these (Burst 2 – Burst 1) to see how their performance changed across time. On average we found a 0.16s improvement in reaction times (*M* = −0.16, 95% CI: [−0.20, −0.12]), which was significantly different from 0 (*t* = −7.46, *df* = 170, *p* = 4.28e−12). This would suggest that on average participants got substantially faster in their reaction times in a year’s time (between the two bursts) on this task. However, analysis based on these simple aggregates is confounded by practice effects. Next, we discuss how fitting the BDEM to this data showed different results.

#### Group-level (population) estimates and individual differences in the asymptote, change of asymptote, learning rates and intra-individual variability parameters based on the Bayesian double exponential model

**FIGURE 8:**
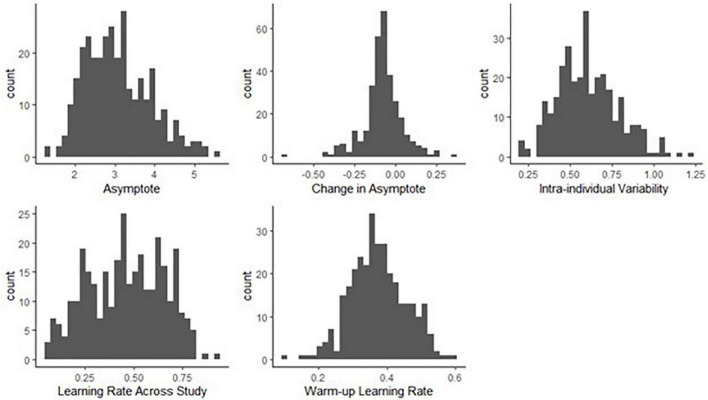
Histograms of person-specific estimates for key BDEM parameters based on data from the symbol search task. The horizontal axis shows the (binned) parameter values while the vertical axis displays the frequency of occurrence of that value among participants.

We found a considerable amount of individual variation in asymptote, change of asymptote, learning rates and intra-individual variability parameters. Figure 8 shows the distributions of the person-specific point estimates of these parameters. Correspondingly, Table 1 shows their group-level averages (population mean estimates, e.g., β_*a, int*_ for asymptote) and the amount of individual differences in them (heterogeneity in terms of population standard deviation estimates, e.g., σ_*a*_, for asymptote). The column labeled “Mean” displays a point estimate for these parameters based on their posteriors, while the column labeled “PSD” shows the corresponding standard deviation around this point estimate, quantifying standard error.

**TABLE 1:** Group-level (population) estimates of Bayesian double exponential model (BDEM) parameters based on data from the symbol search task.

Process parameter	Mean	*PSD*
Asymptote averaged across individuals	2.83	0.06
Heterogeneity in asymptote (SD)	0.75	0.04
Change in asymptote averaged across individuals	−0.07	0.03
Heterogeneity in change in asymptote (SD)	0.19	0.02
Intra-individual variability averaged across individuals	0.56	0.02
Heterogeneity in intra-individual variability (SD)	0.18	0.01
Learning rate across study, averaged across individuals	0.49	0.04
Heterogeneity in learning rate across study (SD)	0.27	0.02
Warm-up learning rate averaged across individuals	0.39	0.05
Heterogeneity in warm-up learning rate (SD)	0.14	0.03

PSD indicates posterior standard deviation of the estimates, which quantifies standard error.

We can see that on average, the asymptote (i.e., peak performance, β_*a*, *int*_) was 2.83 s (*M* = 2.83, *PSD* = 0.06) on this task. This intercept value (and the ones below) is the across person average corresponding to a participant who does not have the MCI status, who is male, and whose age and years of education are at the sample mean level. There was considerable between-person variability in the asymptote, as shown by the standard deviation estimate (*M* = 0.75, *PSD* = 0.04) and the histogram of the person-specific asymptote estimates (first plot of Figure 8).

The (across-person) average difference in peak performance (asymptotes) between the first and the second bursts (β_△, *int*_) was −0.07 s (again, this corresponds to a participant who does not have the MCI status, who is male, and whose age and years of education are at the sample mean level), and it was credibly negative (*M* = −0.07, *PSD* = 0.03). This suggests that even when the retest effects were accounted for, there was an improvement in reaction time performance across bursts. However, the individual differences were considerable, as shown in the second plot of Figure 8: for example, for some participants, there was actually a slowing in reaction times, as shown by their positive change in peak performance estimate. In Section “Person-specific inference on the change in asymptotic performance via Bayesian probability distributions.,” we will show how we can further scrutinize these individual-level estimates to get a probability estimate on whether the detected change represents credible decline in cognitive performance. Finally, we also note that 171 participants did not have second burst data yet, therefore their change estimates were informed by the population mean so they were all concentrated around −0.07.

The average intra-individual variation in RT (β_σ_*e*_, *int*_) was 0.56 s (*M* = 0.56, *PSD* = 0.02), with a large amount of variation across participants, quantified by the group-level standard deviation of the intra-individual variation parameter (*M* = 0.18, *PSD* = 0.01) and illustrated in the third plot of Figure 8. This suggests that individuals differ from each other considerably in terms of how much their cognitive performance fluctuates across the days.

Finally, with respect to the learning rate, a quick visual assessment of the plots in the second row of Figure 8 reveals that person-specific learning rates across study (between bursts) tend to be somewhat higher than the within-burst (warm-up) learning rates (see also corresponding entries in Table 1: *M* = 0.49 vs. *M* = 0.39); however, we can again see considerable amount of individual differences. Next, we look at the results of regressing these parameters on predictors to identify the sources of the individual differences.

#### Explaining sources of individual differences in the asymptote, change of asymptote, and intra-individual variability parameters with the Bayesian double exponential model

The person-specific asymptote, change in asymptote, intra-individual variability, learning rate across study and warm-up learning rate parameters were regressed on predictors quantifying age at baseline (standardized to mean of 0 and standard deviation 1), MCI status (coded as 0 and 1), sex (with 0 for female and 1 for male) and years of education (standardized similarly). Reported effects of age and education were all corresponding to 1 SD unit increase (4.83 years for baseline age, 3.55 years for years of education). Results on the regression coefficients quantifying their associations are summarized in Table 2. Just like in Table 1, the column labeled “Mean” displays a point estimate for these parameters, while the column labeled “PSD” shows the corresponding standard error. The last two columns show the probability that the regression coefficient is below and above 0, respectively, based on the posterior probability mass. For a credible effect we want to see at least 95% (0.95) probability of being either entirely below 0 or entirely above 0. However, we will also discuss if there was moderate evidence for effects, defined as at least 90% (0.9) probability of being either entirely below 0 or entirely above 0 (but not reaching the threshold of 0.95 for credible effect).

**TABLE 2:** Summary of links between cognitive performance characteristics of the symbol search task and selected explanatory variables.

Process parameter	Predictor	Mean	*PSD*	<0	>0
Asymptote	Age	0.11*	0.05	0.01	0.99
	MCI status	0.73*	0.10	0.00	1.00
	Sex	0.03	0.10	0.39	0.61
	Years of education	−0.12*	0.05	1.00	0.00
Change in asymptote	Age	0.03^	0.02	0.06	0.94
	MCI status	−0.03	0.05	0.74	0.26
	Sex	0.01	0.04	0.37	0.62
	Years of education	−0.02	0.02	0.77	0.23
Intra-individual variability	Age	0.01	0.01	0.25	0.75
	MCI status	0.15*	0.03	0.00	1.00
	Sex	0.01	0.02	0.34	0.66
	Years of education	−0.04*	0.01	1.00	0.00
Learning rate across study	Age	0.01	0.02	0.38	0.62
	MCI status	−0.06	0.05	0.88	0.12
	Sex	−0.04	0.05	0.82	0.18
	Years of education	−0.01	0.02	0.68	0.32
Warm-up learning rate	Age	0.05*	0.03	0.03	0.97
	MCI status	−0.06	0.05	0.88	0.12
	Sex	0.01	0.05	0.41	0.59
	Years of education	−0.03	0.03	0.89	0.11

Estimates with an * are meaningfully different from zero (at least 95% probability of being either entirely above or below 0). Estimates with a ^ denote moderate evidence for an effect (at least 90% probability of being either entirely above or below 0). SD indicates posterior standard deviation of the estimates. Column “<0”/“>0” displays the probability of the parameter being smaller/larger than 0.

The first part of Table 2 shows that individual differences in asymptote (peak performance) were credibly linked to age, MCI status and years of education. With older age at baseline, peak performance reaction times showed credible slowing (0.11 s for each standard deviation of age). With positive MCI status there is also on average a 0.73 s slower peak performance reaction time. In contrast, with more years of education, peak performance reaction times tended to be faster [0.12 s faster per 1 SD (3.5) increase in years of education]. We did not find evidence for differences based on sex.

The second section of Table 2 summarizes the results with respect to changes in peak performance over time – that is between the two bursts in the study that were separated on average by a year. We only found trending support for association between age and change in peak performance: for each additional year older at baseline, participants tended to show a 0.03 s slowing of peak reaction times across the two bursts. For this effect to be credibly different from 0 there was 0.94 probability, which is slightly below our 0.95 threshold for credible effect. None of the other predictors showed credible links with this parameter.

The third section of Table 2 shows that differences in intra-individual variability in performance across time were credibly linked to MCI status and years of education. With positive MCI status there was a 0.15 s increase in the variability (in standard deviation units), while participants with 1 SD increases in years of education tended to show 0.04 s less variation.

The last two sections of Table 2 summarize the links between the learning rate parameters (across study and warm-up) and the selected predictors. We found only one credible link: participants with older age at baseline tended to show faster warm-up rate, meaning that they reached their peak performance faster in the second burst (0.05 s faster per one standard deviation on change in age).

### The grid memory task

We did an initial data exploration for the grid memory task−much like we did for the symbol search task−by comparing differences in manifest performance between Burst 1 and Burst 2. We created person-specific difference scores between Burst 1 and Burst 2 averages based on the error distance measure. Across participants we found an improvement across bursts, specifically 0.21 units less error (*M* = −0.21, 95% CI: [−0.26, −0.15]), which was significantly different from 0 (*t* = −7.72, *df* = 170, *p* = 9.404e−13). This would suggest that participants’ memory performance improved in a year’s time (between the two bursts) on this task. However, as before, these simple aggregates are confounded by practice effects. We discuss results from the BDEM next.

#### Group-level estimates (population) estimates and individual differences in the asymptote, change of asymptote, learning rates, and intra-individual variability parameters based on the Bayesian double exponential model

**FIGURE 9:**
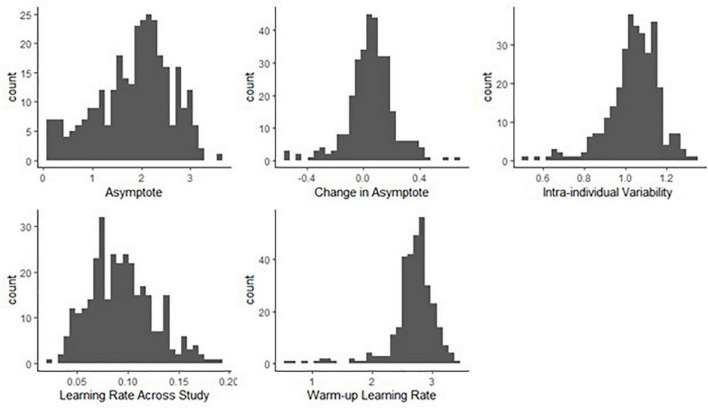
Histograms of person-specific estimates for key BDEM parameters based on data from the grid memory task. The horizontal axis shows the (binned) parameter values while the vertical axis displays the frequency of occurrence of that value among participants.

Similar to the symbol search task, we found considerable amount of individual variation in asymptote, change of asymptote, learning rates and intra-individual variability parameters. Figure 9 shows the distributions of the person-specific point estimates of these parameters. Correspondingly, Table 3 shows their group-level averages and the amount of individual differences in them (following the same logic as in Table 1).

**TABLE 3:** Group-level (population) estimates of BDEM parameters based on data from the grid memory task.

Process parameter	Mean	*PSD*
Asymptote averaged across individuals	1.85	0.09
Heterogeneity in asymptote (SD)	0.69	0.03
Change in asymptote averaged across individuals	0.06	0.04
Heterogeneity in change in asymptote (SD)	0.25	0.03
Intra-individual variability averaged across individuals	1.06	0.02
Heterogeneity in intra-individual variability (SD)	0.16	0.01
Learning rate across study, averaged across individuals	0.09	0.03
Heterogeneity in learning rate across study (SD)	0.04	0.01
Warm-up learning rate averaged across individuals	2.67	0.69
Heterogeneity in warm-up learning rate (SD)	0.99	0.23

PSD indicates posterior standard deviation of the estimates, which quantifies standard error.

We can see that on average, the asymptotic, peak performance (β_*a*, *int*_) was 1.85 units of error (*M* = 1.85, *PSD* = 0.09) on this task and that there was considerable between-person variance in peak performance, as shown by the standard deviation estimate (*M* = 0.69, *PSD* = 0.03) and the histogram of the person-specific asymptote estimates (first plot of Figure 9).

The (across-person) average difference in asymptotes (peak performance) between the first and the second bursts (β_△, *int*_) was 0.06 units of error (*M* = 0.06, *PSD* = 0.04). As opposed to credible improvement in peak reaction times on the symbol search task, this represents trending evidence for decline in performance over time. The individual differences in this peak performance change were also considerable, as shown in the second plot of Figure 9: while for most participants there was some level of decline in performance, there were also some whose performance improved across bursts.

The average intra-individual variation (β_σ_*e*_, *int*_) was around 1 grid unit (*M* = 1.06, *PSD* = 0.02), with a large amount of variation across individuals, quantified by the group-level standard deviation of the intra-individual variation parameter (*M* = 0.16, *SD* = 0.01) and illustrated in the third plot of Figure 9. This provided further evidence that participants differ from each other considerably in terms of how much their cognitive performance fluctuates across the days.

Finally, with respect to the learning rate, we found a different pattern than in the symbol search task: in this task the person-specific learning rates across study (between bursts) tended to be much lower than the within-burst (warm-up) learning rates, as illustrated in the second row of Figure 9 (see also corresponding entries in Table 3: *M* = 0.09 vs. *M* = 2.67); however, we can again see large individual differences. We again look at the results of regressing these parameters on predictors to identify the sources of the individual differences next.

#### Explaining sources of individual differences in the asymptote, change of asymptote, and intra-individual variability parameters with the Bayesian double exponential model

The first part of Table 4 shows that individual differences in asymptote (peak performance) were credibly linked to MCI status, sex and years of education. With positive MCI status the peak performance error rates were higher (on average by 0.44 units of error). In contrast, with being male and with more years of education, peak performance error rates tended to be lower (0.44 and 0.26 units of error, respectively). We did not find evidence for differences based on age.

**TABLE 4:** Summary of links between cognitive performance characteristics of grid memory task and selected explanatory variables.

Process parameter	Predictor	Mean	*PSD*	<0	>0
Asymptote	Age	0.01	0.05	0.39	0.61
	MCI status	0.44*	0.10	0.00	1.00
	Sex	−0.44*	0.10	1.00	0.00
	Years of education	−0.26*	0.05	1.00	0.00
Change in asymptote	Age	0.05*	0.03	0.03	0.97
	MCI status	0.05	0.06	0.22	0.78
	Sex	−0.05	0.05	0.84	0.16
	Years of education	−0.04*	0.03	0.95	0.05
Intra-individual variability	Age	−0.03*	0.01	0.99	0.01
	MCI status	0.01	0.03	0.35	0.65
	Sex	−0.08*	0.03	1.00	0.00
	Years of education	−0.02*	0.01	0.96	0.04
Learning rate across study	Age	0.01*	0.10	0.03	0.97
	MCI status	−0.01	0.03	0.82	0.18
	Sex	0.03*	0.02	0.03	0.97
	Years of education	0.02*	0.01	0.01	0.99
Warm-up learning rate	Age	−0.08	0.14	0.73	0.27
	MCI status	0.03	0.86	0.56	0.44
	Sex	−0.01	0.33	0.52	0.48
	Years of education	−0.13	0.17	0.78	0.22

Estimates with an * are meaningfully different from zero (at least 95% probability of being either entirely above or below 0). Estimates with a ^ denote moderate evidence for an effect (at least 90% probability of being either entirely above or below 0). SD indicates posterior standard deviation of the estimates. Column “<0”/“>0” displays the probability of the parameter being smaller/larger than 0.

The second section of Table 4 summarizes the results with respect to changes in peak performance over time – that is between the two bursts in the study that were separated on average by a year. We found credible support for association between age and change in peak performance: participants who were older at baseline tended to show worsening error rates (by 0.05 units of error) across the two bursts. In contrast, with more years of education participants tended to show improvement in error rate over time (0.04 units less). None of the other predictors showed credible links with this parameter.

The third section of Table 4 shows that differences in intra-individual variability in performance across time were credibly linked to age, sex, and education level: with older age, being male, and with more years of education, there was less variability (0.03, 0.08, and 0.02 in standard deviation units, respectively).

The last two sections of Table 4 summarize the links between the learning rate parameters (across study and warm-up) and the selected predictors. We found credible links only with across study learning rates, but the effect sizes were low. Older age at baseline, males, and participants with more years of education tended to be faster across study learning rates.

### Person-specific inference on the change in asymptotic performance via Bayesian probability distributions

As stated before, the result of the Bayesian inference is a posterior probability distribution for every model parameter. Based on these distributions, probabilities on different ranges of the parameters can be calculated. This means, for example, decisions on the “significance” of regression effects do not need to be binary with an implausible null hypothesis of absolutely no difference. Instead, we can just make an informed decision by looking at the posterior probability distribution of the regression coefficient.

**FIGURE 10:**
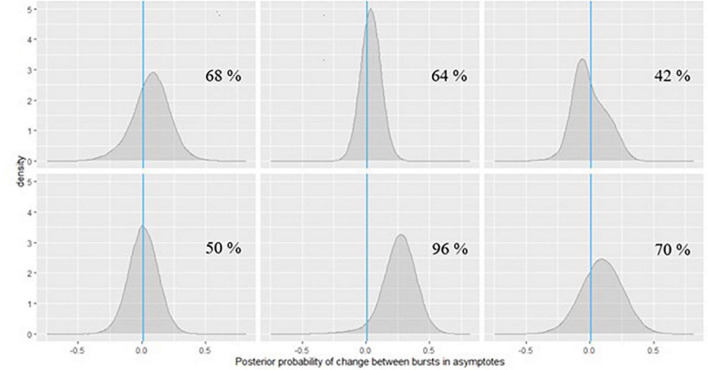
Posterior probabilities of change in the symbol search task performance for 6 EAS participants.

**FIGURE 11:**
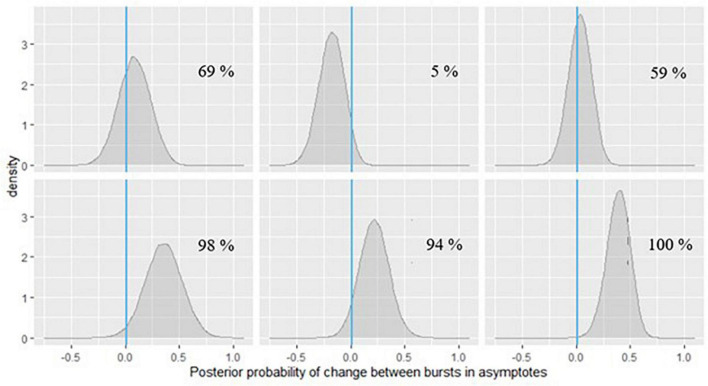
Posterior probabilities of change in the grid memory task performance for 6 EAS participants.

Inference can be done similarly for the person-specific parameters which are likely indicators of dementia risk, as on the change in peak performance across bursts. An example is shown in Figure 10 for symbol search and Figure 11 for grid memory data featuring the same six example participants as in Figures 6, 7. We can decide based on theoretical arguments whether a less than 0.01 s difference in peak performance (or 0.01 unit of error) represents a practically relevant effect. Using Monte Carlo integration, we can then calculate how much of the posterior mass falls above 0.01 (indicated with a vertical line in Figures 10, 11) – resulting in the probability of a practically relevant decline based on the participant’s change in performance on a particular task between two bursts.

In Figure 10, we can see that for the participants in the first row and the first one in the second row, the posterior probabilities do not provide much evidence for practically relevant change – it is approximately the same amount of probability mass on both sides of 0. However, for the participant in the second plot of the second row of Figure 10, there is a 96% chance of such decline in symbol search performance, and the magnitude of decline is around 0.25 s, based on the peak of the posterior distribution (a more accurate point estimate can also be calculated). If we check the same participant’s change in peak performance estimate from the grid memory task in Figure 11, there is a 94% probability of decline there, with the magnitude of decline being a bit less than 0.25 units of error, based on the peak of the posterior distribution. Inferences like this could be drawn for every person to evaluate their individual dementia risk.

As can be seen in Figure 11, the participants in the last row show high probabilities of cognitive decline based on their grid memory performance across the two bursts. For the participant in the third plot of the second row, there was already some support for decline on the symbol search task (70% chance, see Figure 10). Numerical probability estimates could also be combined together in a predictive modeling framework for efficient inference.

## Discussion

### Peak performance and changes in peak performance across bursts

In our analyses above, we aimed to isolate peak performance from retest effects in repeated measures of cognitive performance. We found that individual differences in the peak performance estimates were meaningfully related to the selected predictors. For example, MCI status was linked to decreased peak performance in both tasks.

When we explored the grid memory data by comparing burst averages, we found significant improvement across bursts. In contrast, the BDEM showed moderate evidence for decline in cognitive performance across bursts on this task. This suggests that disentangling learning processes from other latent cognitive changes is critical for this type of data. Individual differences in the change in peak performance across bursts were plausibly related to age (more error) and education (less error), further supporting the usefulness of our approach.

In contrast, on the symbol search task there was a 70-ms improvement across bursts in peak performance RT, even when retest learning effects were taken into account with the BDEM. However, this improvement is still smaller than the difference in burst averages (160 ms improvement), indicating that some retest learning was indeed accounted for by the BDEM. There are several possible reasons why we found improvement in peak RT on this task. It could be partly because we only have two bursts to examine change in peak performances across the years, so that we might not have had enough information to accurately capture the change process. Another reason for improvement in RTs on the symbol search task could be related to the fact that we were only modeling RTs from correct trials. Modeling all RTs in combination with accuracies for example in a drift diffusion model framework (see, e.g., Wagenmakers, 2009) could provide more insight.

### Within-person variability in performance across days

While variability in performance is generally acknowledged in repeated assessments of cognitive performance, it is treated most often as a nuance. In the current study, we found that in the symbol search data, participants with MCI status showed more variability across days in their reaction times. Also, consistently across the two tasks, individuals with more years of education exhibited less variability. Paired with our previous findings that intra-individual variation in performance predicts MCI status, this may suggest that day-to-day variation reflects individual differences in cognitive reserve (Cerino et al., 2021).

### Learning rates across study and within a burst

Learning effects confound the detection of cognitive change by biasing estimates of the underlying performance on a given assessment. In our study, we distilled these from peak performance estimated, but also considered them as potential indicators of cognitive change/decline given age- and disease-related impacts on brain subsystems that support learning. We extracted features of short- and long-timescale learning/retention in terms of within-burst or warm-up learning rate and across the study learning rate. On the symbol search task, we only found limited evidence (88% probability) of individuals with MCI status exhibiting slower learning; however, this effect was consistent for across study and warm-up learning rates (see Table 2). Surprisingly, on this task the only credible link was between learning and age, where participants who were older at baseline tended to show faster warm-up learning rate. Similar credible age effect was found in the grid memory data as well, although the effect was small and these participants also tended to have worse peak performance, therefore the steep learning might not indicate better brain health in this context.

### Limitations and future directions

The double negative exponential model applied to measured burst data has the potential to provide a significant contribution toward accurately detecting and quantifying cognitive decline by disentangling practice effects from latent indicators of cognitive performance (i.e., asymptotic performance). It also provides clinically useful information in terms of personalized probabilities of impairment and decline for every individual, which can be useful to a clinician. We see several extensions of the BDEM approach for future projects. First, the BDEM parameter estimates on different tasks could be compared in terms of their predictive performance of neurodegenerative diseases. The goal is to optimize a model that has several of the key BDEM parameters as indicators, potentially from various cognitive domains (i.e., using more than one type of cognitive task). Second, while the current analysis did not yield promising results on linking learning process parameters with MCI status, it is possible that further exploration with indicators that are more specific to ADRD (such as blood biomarkers) could provide more insight. This is particularly relevant given that classification of MCI is a heterogenous classification, which as we highlighted in the introduction can have limited reliability. Finally, the BDEM could be combined with cognitive process models, such as the drift diffusion model that breaks down performances to meaningful cognitive characteristics. Combining such a drift diffusion modeling approach with the BDEM would allow us to simultaneously model and map learning features (e.g., learning rate) and changes in peak performance (and all associated random effects) onto cognitive (drift rate), and meta-cognitive (boundary separation) parameters.

## Data availability statement

The data analyzed in this study is subject to the following licenses/restrictions: Interested collaborators are asked to complete a concept proposal form (details for potential project, paper, or abstract) to be reviewed and forwarded to the Einstein Aging Study Steering Committee for consideration. Requests to access these datasets should be directed to MK, MPH at mindy.katz@einsteinmed.org. For additional information on data sharing requests for the Einstein Aging Study, see https://www.einsteinmed.edu/departments/neurology/clinical-research-program/eas/data-sharing.html.

## Ethics statement

The studies involving human participants were reviewed and approved by the Institutional Review Board at Albert Einstein College of Medicine. The patients/participants provided their written informed consent to participate in this study.

## Author contributions

ZO, JH, and MS conceptualized the theoretical model and research questions. ZO led the writing of the manuscript and conducted the data analysis. MS, MK, and CW designed the original study and supervised the data collection. All authors contributed to the writing of the manuscript, provided feedback on the data analysis, and made critical revisions of the manuscript for intellectual content.

## Footnotes

1
https://osf.io/h9yqk/?view_only=9e311fca177e462bbdb347c50736ae21

2 Additionally, plots showing the raw data averaged across persons and the corresponding model fits for both the symbol search task and dot memory task can be found on the above referenced OSF site. These plots showed satisfactory fit of the BDEM on the group level.
